# Screening and identification of genes related to ferroptosis in keratoconus

**DOI:** 10.1038/s41598-023-41194-2

**Published:** 2023-08-25

**Authors:** Xiaojun Wu, Qing Deng, Zhe Han, Feixue Ni, Daxi Sun, Yuxue Xu

**Affiliations:** 1 School of Pharmacology, Binzhou Medical University, Guanhai Rd 346, Yantai, 264003 China; 2 Shandong Technology Innovation Center of Molecular Targeting and Intelligent Diagnosis and Treatment, Yantai, 264003 China

**Keywords:** Biomarkers, Gene expression

## Abstract

Corneal keratoconus (KC) is a dilated (ectatic) corneal disease characterized by a central thinning of the cornea, which causes protrusion into a conical shape that seriously affects vision. However, due to the complex etiology of keratoconus, its entire mechanism remains unclear and there is no mechanism-directed treatment method. Ferroptosis is a novel programmed cell death mechanism related to lipid peroxidation, stress, and amino acid metabolism, which plays a crucial role in various diseases. This study aimed to explore the relationship between keratoconus and ferroptosis, to provide new insights into the mechanism of keratoconus development, and potential treatment options based on further elucidation of this mechanism. The corresponding mRNA microarray expression matrix data of KC patients were obtained from GEO database (GSE204791). Weighted co-expression network analysis (WGCNA) and support vector machine recursive feature elimination (SVM-RFE) were selected to screen hub genes, which were overlapped with ferroptosis genes (FRGs) from FerrDb. GO and GSEA were performed to analyze differential pathways, ssGSEA was used to determine immune status, and then, feasible drugs were predicted by gene-drug network. Additionally, we predicted the miRNA and IncRNA of hub genes to identify the underlying mechanism of disease so as to predict treatment for the disease. The epithelial transcriptome from keratoconus tissue mRNA microarray data (GSE204791) was extracted for the main analysis, including eight epithelial cells and eight epithelial control cells. The differential genes that were overlapped by WGCAN, SVM-RFE and FRGs were mainly related to oxidative stress, immune regulation, cellular inflammation, and metal ion transport. Through further analysis, aldo–keto reductase family 1 member C3 (AKR1C3) was selected, and negatively correlated with mature CD56 natural killer (NK) cells and macrophages. Then, gene-drug interaction network analysis and miRNA prediction were performed through the website. It was concluded that four immune-related drugs (INDOMETHACIN, DAUNORUBICIN, DOXORUBICIN, DOCETAXEL) and a miRNA (has-miR-184) were screened to predict potential drugs and targets for disease treatment. To our knowledge, this was the first report of KC being associated with ferroptosis and prompted search for differential genes to predict drug targets of gene immunotherapy. Our findings provided insight and a solid basis for the analysis and treatment of KC.

## Introduction

Keratoconus is a degenerative corneal disease characterized by progressive corneal thinning, protrusion of the cornea and progressive irregular astigmatism, which can result in corneal fibrosis and visual deterioration^1,2^. Keratoconus usually occurs during adolescence, with a clinical course extending over 10 or 20 years, potentially advancing to an advanced stage^3^. Keratoconus was previously thought to result from non-inflammatory environmental^4^, metabolic, and genetic factors^5^. In the early stages, disease intervention in keratoconus can be achieved by wearing contact lenses to correct irregular astigmatism^6^. However, with further disease progression, approximately 20% of advanced patients can only recover vision by penetrating keratoplasty^6,7^. At present, keratoconus corneas are principally treated either medically or with surgery (bowman layer transplantation^8^, or corneal cross-linking approaches^9^).

There is increasing evidence that the pathogenesis of keratoconus is affected by a variety of other factors, such as metabolism^1^, immune response^10,11^, and oxidative stress^12^. Previous reports have shown that keratoconus corneas seem to be positively associated with immune-mediated disease, and systemic inflammatory response may play a role in its pathogenesis. Through analysis of ocular surface immune cells in 51 KC and 15 healthy controls, Ghosh et al.^13^ identified a unique immune-inflammatory component as well as its potential as an additional therapeutic target in the treatment of KC. Moreover, oxidative stress reportedly has induced autophagy dysregulation in corneal epithelial cells of keratoconus patients, from a study that demonstrated expression levels of autolysosomal pathway markers in corneal epithelial cells from patients with KC and normal human corneal epithelial (HCE) cells under oxidative stress. Autophagy, induced by oxidative stress, may be a key factor in the pathogenesis of KC^12,14^.

Ferroptosis, as a relatively newly-described type of cell death, the inducing factors of which affect glutathione peroxidase through different pathways, both directly or indirectly, result in decreased antioxidant capacity which allows reactive oxygen species to accumulate^15–17^. The process by which ferroptosis occurs has been closely associated with immune regulation, inflammatory regulation and mitochondrial metabolism of cells^18,19^, and strong evidence has linked ferroptosis with the diagnosis and treatment of multiple diseases including cancers (PAAD^20^, CA^21^, COAD^22^), diabetes complications (DN^23^, DR^24^), and cardiovascular diseases^25^.

Controversy continues regarding the overall pathogenesis of keratoconus, and its treatment options have also been limited pending clarification of the relationship between keratoconus and reactive oxygen species, inflammation and immune metabolism. BUDD et al. found that levels of oxygen-derived free radicals were higher in the KC cornea than in the normal cornea^26^. Moreover, Hao et al. demonstrated that ROS and RNS could drive oxidative damage to mitochondrial DNA and affect the oxidative phosphorylation pathway, which resulted in blockage of collagen synthesis in the extracellular matrix^27^. Therefore, we speculated that ferroptosis might provide new, valuable information in the pathogenesis of KC.

Analyzing genome-wide transcriptome data provides a powerful tool to investigate associations between gene expression and traits^28^. In this way, hub genes and therapeutic targets for diseases can be predicted. In the present study, bioinformatics analysis was performed using EKC and EN transcriptome data from GSE204791. Then, WGCNA and SVN-RFE were used to screen core modules that overlapped hub genes with ferroptosis genes. Next, GO analysis was used to determine gene molecular function. Through research of gene-immune correlations and gene-drug targets, our aim was to predict the possible therapeutic targets of KC. The hub gene AKR1C3 (aldo–keto reductase 1, member C3) was screened by bioinformatics analysis, and its expression was reduced, which meant that it was a ferroptosis inhibitor. We then analyzed AKR1C3-related miRNA and IncRNA to predict gene therapy and immune target drugs for KC. Ultimately, this study provided new insights into KC pathogenesis and a basis for immunotherapy of the disease.

## Materials and methods

### Microarray data acquisition

**Figure 1:**
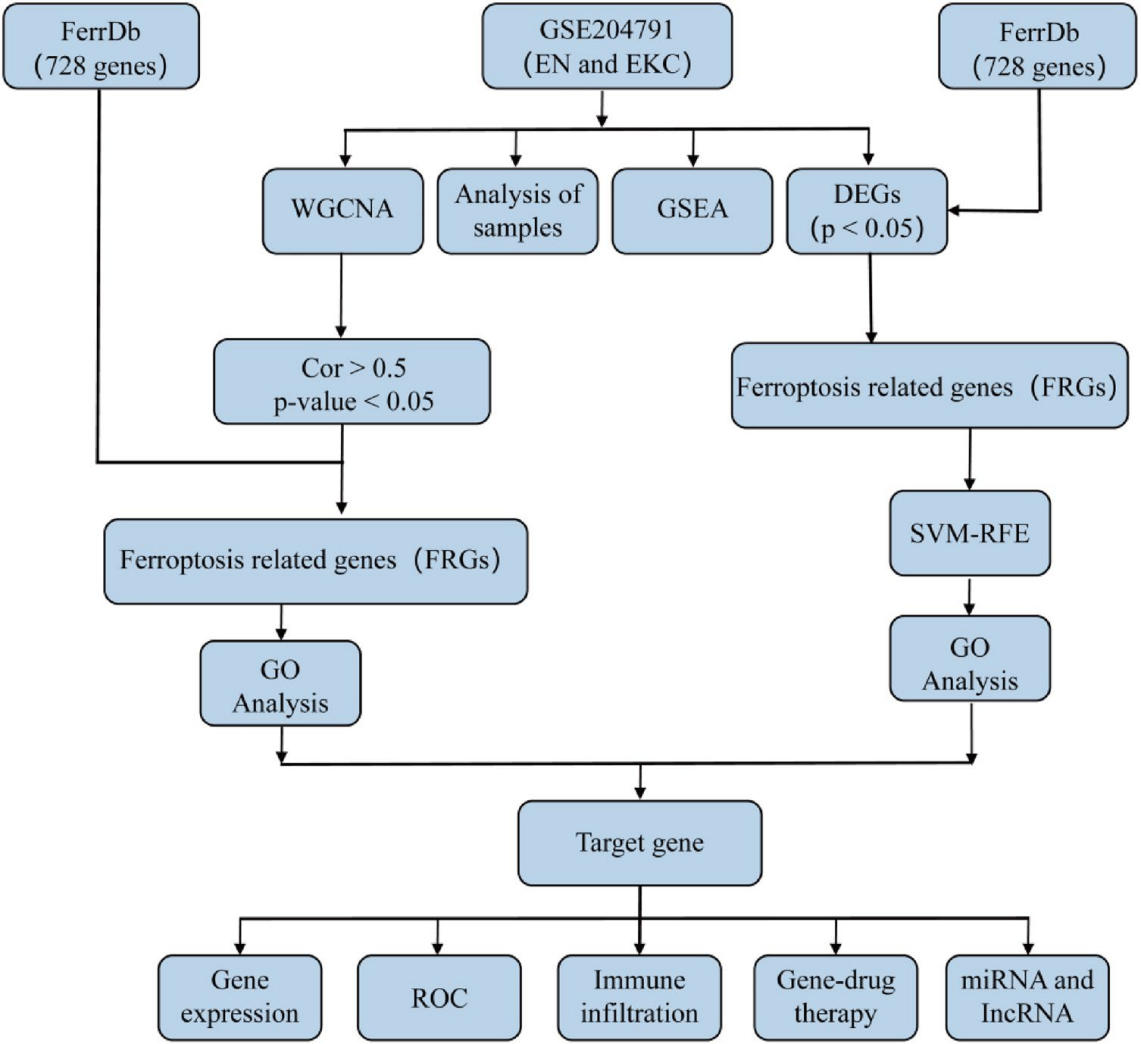
The workflow chart in this study.

We searched the keywords ("keratoconus" AND "Homo sapiens" [porgn:__txid9606]) and "Expression profiling by array" using the GEO database (https://www.ncbi.nlm.nih.gov/geo/) to obtain mRNA Microarray Gene Expression Matrix of Keratoconus. We obtained GEO profiles (GSE204791) which included epithelial cells (8 EN, 8 EKC) and stromal cells (7 SN, 8 SKC) of keratoconus. The GSE204791 dataset matrix is a public database based on platform document GPL21185 (Agilent-072363 SurePrint G3 Human GE v3 8 × 60 K Microarray 039494 [Probe Name Version]), the patients involved in the database had previously given ethical approval, so our study did not involve ethical issues and other conflicts of interest. The workflow is shown in Fig. 1.

### Data stability verification

The dist function was used to calculate the distance between each data category; the R package (ggplot) was executed for visual analysis of the data to make a clustering dendrogram. In addition, we performed dimensionality reduction on the data, and used the ggplot (3.3.6) package to apply visualization to the PCA analysis of the data.

### Gene set enrichment analysis

The gene set enrichment analysis (GSEA) was used to evaluate the distribution trend of the defined gene set in the gene table ranked relative to the phenotype, to judge its contribution to the phenotype. Gene expression data in GSE204791 was analyzed with the use of the c2.cp (C2.cp.v2023.1hs. Symbols. gmt) collection, and the Molecular Signature Database (MSigDB) served as a reference gene set. Gene set data were included in the GSEA software (version 4.3.2), and item significant enrichment criteria: *p* value < 0.05.

### Construction of weighted gene co-expression network analysis

Weighted Gene Co-Expression Network Analysis was performed using R package (WGCNA). The “limma” package normalized the data and used Pearson to cluster the samples and average linkage analysis, delete small fluctuations and missing values; the adjacency matrix was transformed into a TOM matrix and genes were then clustered. The screened groups were represented by different colors, and the gene set (*p* < 0.05, correlation ≥ 0.5) were overlapped with the ferroptosis database (http://www.zhounan.org/ferrdb/current/); there were 728 genes involved in ferroptosis, including driver genes, suppressor genes and marker genes.

### Gene enrichment analysis

To obtain the function and biological process of ferroptosis-related differential mRNA, the data were annotated by gene ontology (GO) using the "go plot", "cluster profiler", and "circlize" package in R version (4.3.2), and then the ggplot2 package was used for visualization.

### Support vector machine-recursive feature elimination (SVM-RFE)

Support vector machine (SVM) is a monitoring machine learning technique widely used in classification and regression analysis, and the best genes were selected from the metadata cohort using the RFE algorithm. In conclusion, to identify gene sets with the greatest discriminative power, SVM-recursive feature elimination (SVM-RFE) was utilized to screen for suitable features.

### Single sample gene set enrichment analysis (ssGSEA)

The ssGSEA is based on 28 immune subtypes and contains different genes for immune cell types, functions, pathways, and checkpoints. Different R packages ("limma", "GSEABase", "GSVA") were used to evaluate immune cell properties in different samples, and the results were visualized by using "pheatmap" and "vioplot" packages.

### Gene expression analysis

The gene expression matrix was extracted from the transcriptome samples of GSE204791 using R (4.3.2). And then, the ggpubr package was used to visualize the gene expression levels of AKR1C3 and SLC7A11. A significant difference was considered when *p* < 0.05. The samples for data analysis are the transcriptome samples based on GSE204791, and the dataset matrix is a public database based on the platform file GPL21185. Therefore, the use and analysis of this data do not involve medical ethical review and other conflicts of interest.

### Gene-drug analysis network

The gene-drug network was predicted using the website (https://dgidb.genome.wustl.edu/). The predicted results were based on drugs that target genes reported in previous studies, and a total of 10 different drugs were predicted, including 4 immunomodulatory drugs.

## Results

### Significant signaling pathways were screened by GSEA

Data were obtained from keratoconus epithelial cells (KEC) and epithelial basal cells (EN) in GSE204791, and SKC and SN were used for partial validation. The results of the box-plot (Supplementary Fig. S1A) showed that the black lines on the medial axis of all samples were roughly at the same level, and, therefore, the standardization degree of the samples was satisfactory. The PCA results also confirmed the replicability of the data (Supplementary Fig. S1B). Similarly, we obtained the same results in SKC and SN (Supplementary Fig. S2A and B). In sample processing, we deleted the stromal cell sample GSM6193943, because the sample deviated greatly and did not have satisfactory repeatability characteristics. Next, we performed a cluster tree analysis on the 14 samples using the class-averaging method of hierarchical clustering-Euclidean clustering, and the clustering dendrogram confirmed the clustering relationship between each group (Supplementary Fig. S1C). Our purpose was to illustrate that GSE204791 sample data were reliable and could be used for further analysis. There were 25,964 genes displayed in the KEC and EN samples, and 362 genes with significant differences (*p* value < 0.05, |Log_2_FC (Fold Change)|> 0.58), of which 156 were significantly up-regulated (indicated in red), and 206 were significantly down-regulated (indicated in blue) (Supplementary Fig. S1D).

### GSEA analysis

**Figure 2:**
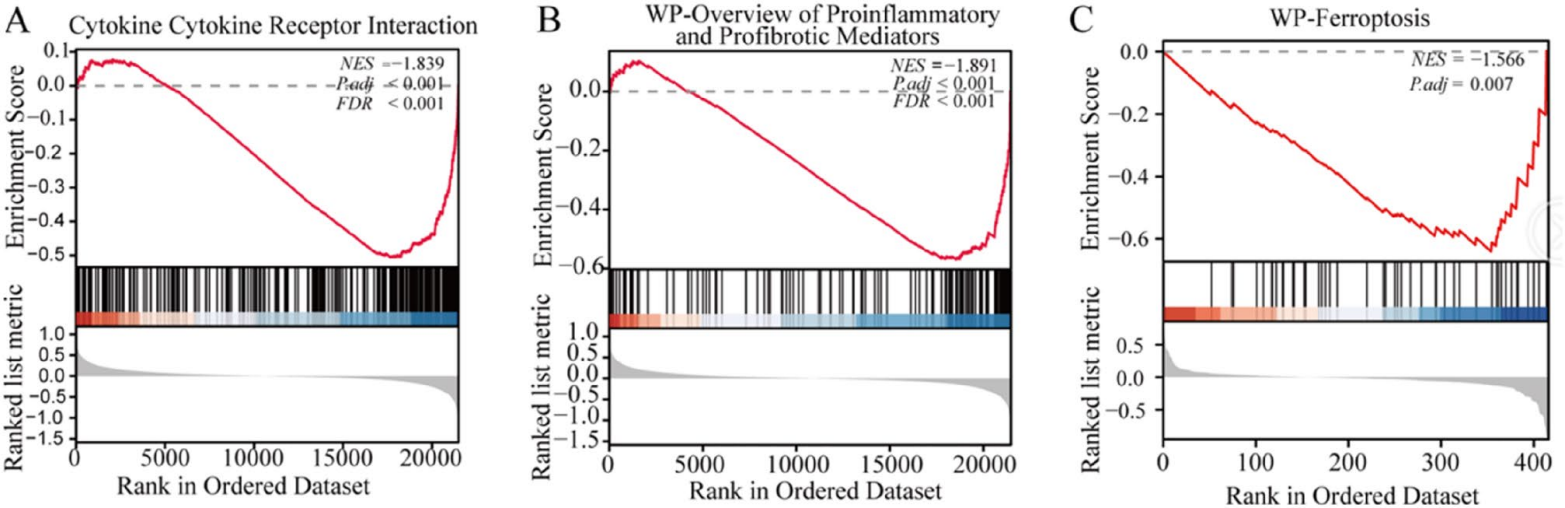
Representative results of GSEA analysis in the expression data of EN and EKC. (**A**) A significant gene set negatively correlated with EKC group was Cytokine-Cytokine Receptor Interaction (NES = − 1.839, P.adj < 0.001). (**B**) Overview of proinflammatory and profibrotic Mediators was negatively correlated with EKC (NES = − 1.567, P.adj < 0.001). (**C**) Ferroptosis-related genes are negatively correlated with keratoconus (NES = − 1.566, P.adj = 0.007). NES, normalized enrichment score.

The gene expression data in GSE204791 were analyzed holistically by GSEA using the C2 (CP) gene sets (MSigDB). As shown in Fig. 2A, the most abundant item identified by GSEA was Cytokine Cytokine Receptor Interaction (p.adj < 0.001), and the enrichment results showed down-regulation. In addition, we found that the gene sets were also gathered in Signaling By interleukin (p.adj = 0.002) (Fig. 2B). Partial GSEA results are provided in Supplementary Table 1. Based on GSEA analysis, the ferroptosis-related gene set was also enriched significantly in the EKC (p.adj = 0.007), and mostly downregulated (Fig. 2C). Similarly, we found the same results in SKC and SN (Supplementary Fig. S3A and B); the ferroptosis-related gene set was enriched significantly, and also mostly downregulated (Supplementary Figure S3C).

### WGCAN screened the hub genes

**Figure 3:**
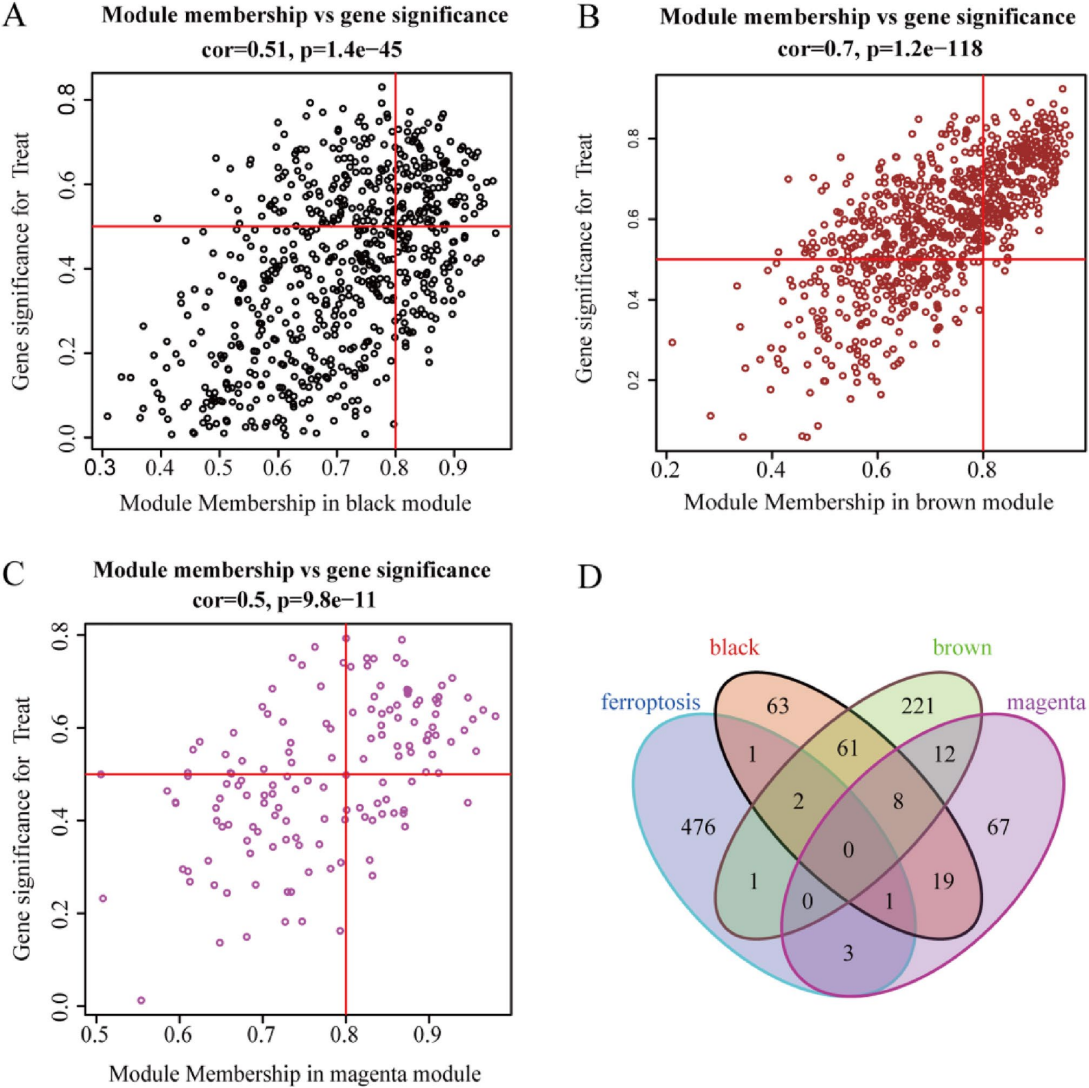
Ferroptosis related genes in the central gene module (*p* < 0.05, cor ≥ 0.5). (**A**-**C**) Gene significance versus Module Membership (MM) in black, brown, and magenta modules. (**D**) Venn diagram screened ferroptosis genes in 3 gene modules.

The hub genes were screened for EKC and EN. We normalized the data and Pearson's correlation coefficient to cluster the samples. WGCNA analysis generally included genes with a false discovery rate < 0.05 and log_2_FC (Fold Change) ≥ 0.5. In our study, the genes with low variability and samples with missing values were identified and removed; all samples were checked and no outlier samples were deleted (Supplementary Fig. S4A). We used the PickSoft-Threshold function in the WGCNA package for analysis. When the soft threshold was 19, the topological relationship of the scale-free network with R2 equal to 0.9 was satisfied, and it had the best connectivity (Supplementary Fig. S4B and C). We constructed gene networks and identified modules using the one-step network building function of the WGCNA R package. The adjacency matrix was converted into TOM matrix, then the number of genes in the dynamic clipping module was set to 60 and the depth segmentation was set to 2 (which implies a medium sensitivity) to construct the WGCNA network (Supplementary Fig. S4D). Finally, 10 modules were successfully constructed, which are represented in 10 different colors (Supplementary Fig. S4E). It is noteworthy that the ME brown (r = 0.82, *p* = 1e−04), ME black (r = − 0.57, *p* = 0.02), and ME magenta (r = − 0.65, *p* = 0.007) modules were significantly correlated with the disease state and may have played an important role in keratoconus (Supplementary Fig. S4F); they were used for further analysis. Furthermore, we also confirmed there was a highly significant correlation between Module membership (Mm) and Gene significance (GS) in these modules (black module: Cor = 0.51, *p* = 1.4e−45 (Fig. 3A); brown module: Cor = 0.7, *p* = 1.2e−148 (Fig. 3B); magenta module: Cor = 0.5, *p* = 9.8–11e (Fig. 3C)). We overlaid these 4 modules with FRGs and found that 8 FRGs (LCE2C, NOS2, AKR1C3, CAV1, MMD, LINC00618, SNCA, SLC7A11) were closely related to the disease state of keratoconus (Fig. 3D).

### SVM—RFE filtering characteristic gene

**Figure 4:**
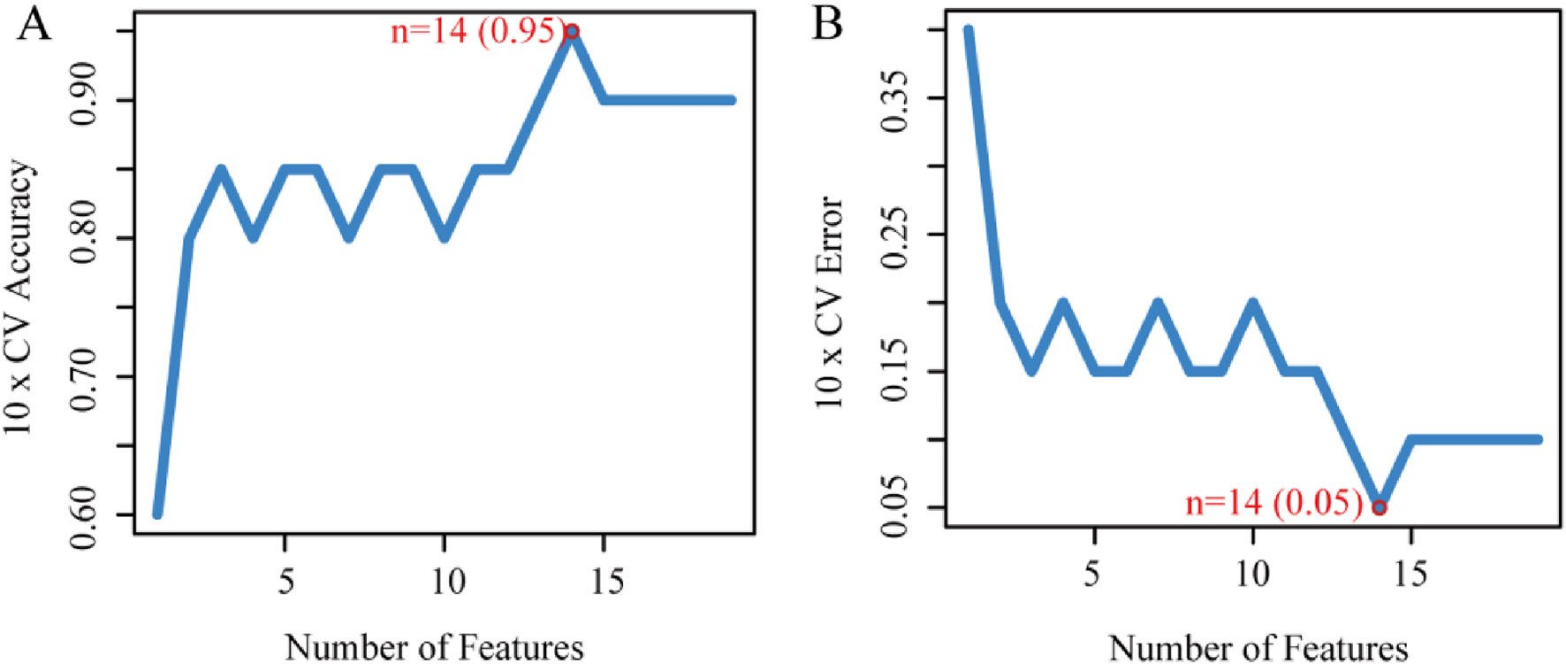
SVM—RFE filtered characteristic genes. (**A**, **B**) When n = 14, 10xCV Accuracy was 0.95, and the classifier had the minimum error (0.05).

Differential genes were screened for EN and EKC and then overlapped with the FRGs gene. The heat map showed there were 89 differential genes associated with ferroptosis (35 up-regulated, 54 down-regulated) (Supplementary Fig. S5). For these 89 genes, the SVM-RFE algorithm was used to screen characteristic genes, and these important genes were ranked for cross-validation. When the number of features was 14 (Fig. 4A,B), the classifier had the minimum error (0.05), the 10xCV Accuracy was 0.95, and 14 genes were selected (RBMS1, AQP5, ATF2, SMPD1, ARNTL, AKR1C3, LCN2, FXN, SLC11A2, BRD7, LCE2C, PARP14, MGST1, MYB).

### GO analysis of ferroptosis genes and screened of target genes

**Figure 5:**
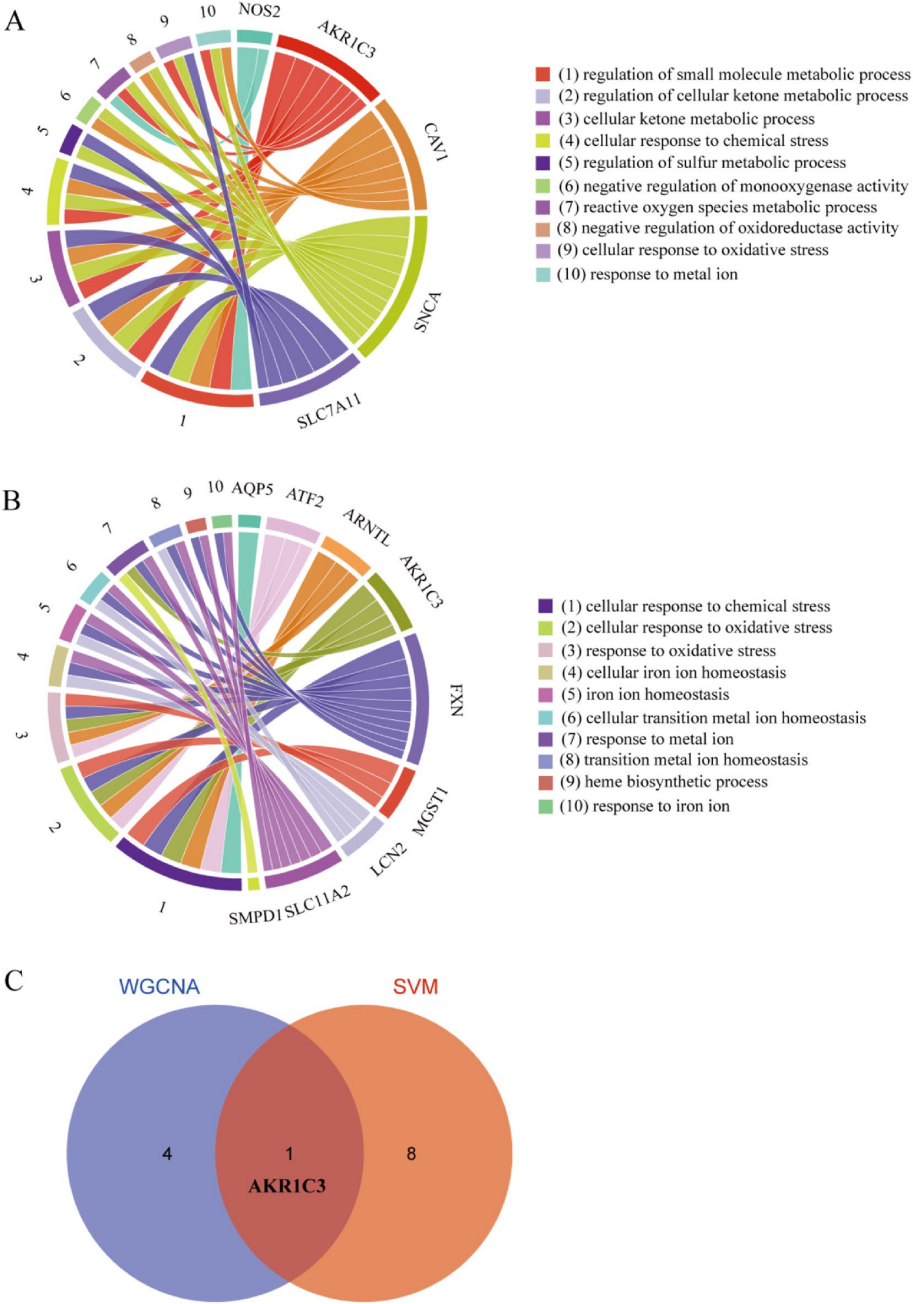
GO analysis of ferroptosis genes. (**A**) GO analysis of differentially expressed genes screened by WGCNA. (**B**) GO analysis of differentially expressed genes screened by SVM—RFE. (**C**) Venn diagram shows intersection genes.

**Figure 6:**
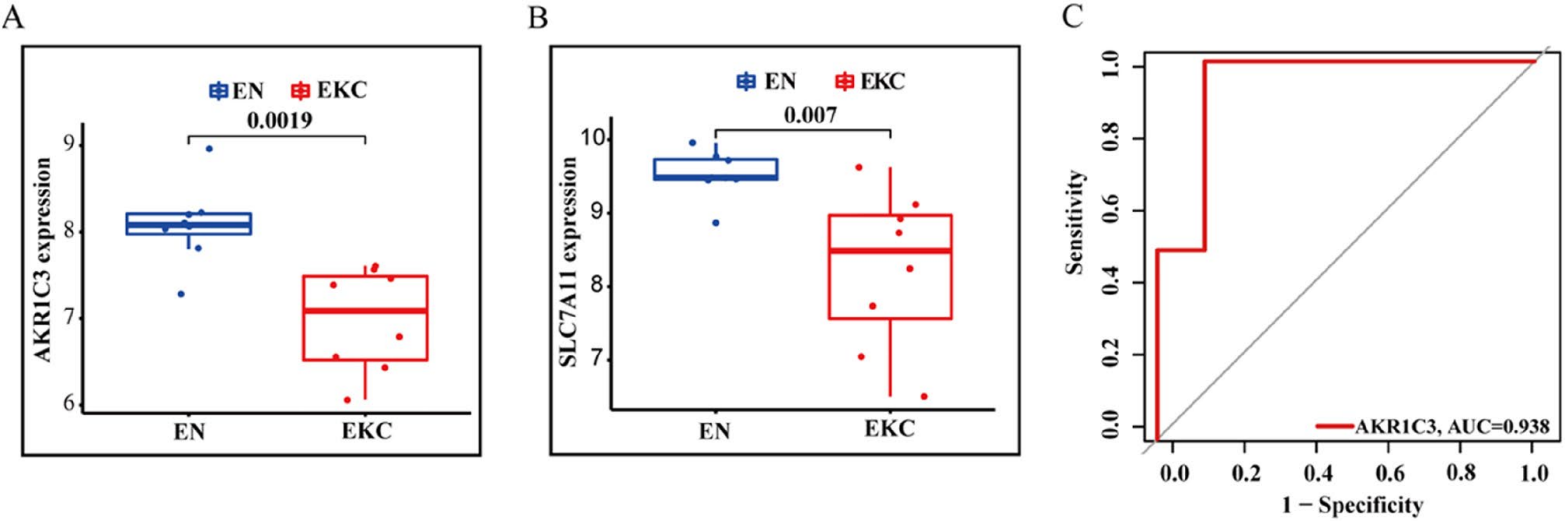
The differential gene expression and Receiver operating characteristic analysis (ROC). (**A**) The mRNA expression of AKR1C3. (**B**) The mRNA expression of SLC7A11. (**C**) The AUC analysis of AKR1C3. Receiver operating characteristic, ROC; AUC, area under the ROC curve.

Eight ferroptosis genes (LCE2C, NOS2, AKR1C3, CAV1, MMD, LINC00618, SNCA, SLC7A11) were selected by WGCNA for GO-BP analysis, and they were significantly enriched in the cellular ketone metabolism process, reactive oxygen species metabolism process, and response to metal ions (Fig. 5A); five genes were involved (NOS2, AKR1C3, CAV1, SNCA, SLC7A11). In addition, ferroptosis genes (RBMS1, AQP5, ATF2, SMPD1, ARNTL, AKR1C3, LCN2, FXN, SLC11A2, BRD7, LCE2C, PARP14, MGST1, MYB) selected by SVM-REF algorithm showed that they were related to the cellular response to oxidative stress and the response to metal ions; nine genes were involved (AQP5, ATF2, ARNTL, AKR1C3, FXN, MGST1, SLC11A2, LCN2, SMPD1) (Fig. 5B). The Venn diagram shows that AKR1C3 is common to them (Fig. 5C). It has important functions in GO-BP and was significantly down-regulated in samples (*p* = 0.0019) (Fig. 6A). Also, the expression of SLC7A11 was significantly reduced in our samples (Fig. 6B). Using univariate analysis of receiver operating characteristic (ROC), the area under the ROC curve for AKR1C3 was 0.938 (Fig. 6C). This further indicated the important predictive performance of AKR1C3 in KC. The same results were obtained in SN and SKC (Supplementary Fig. S6).

### Relationship between core genes and immune microenvironment

**Figure 7:**
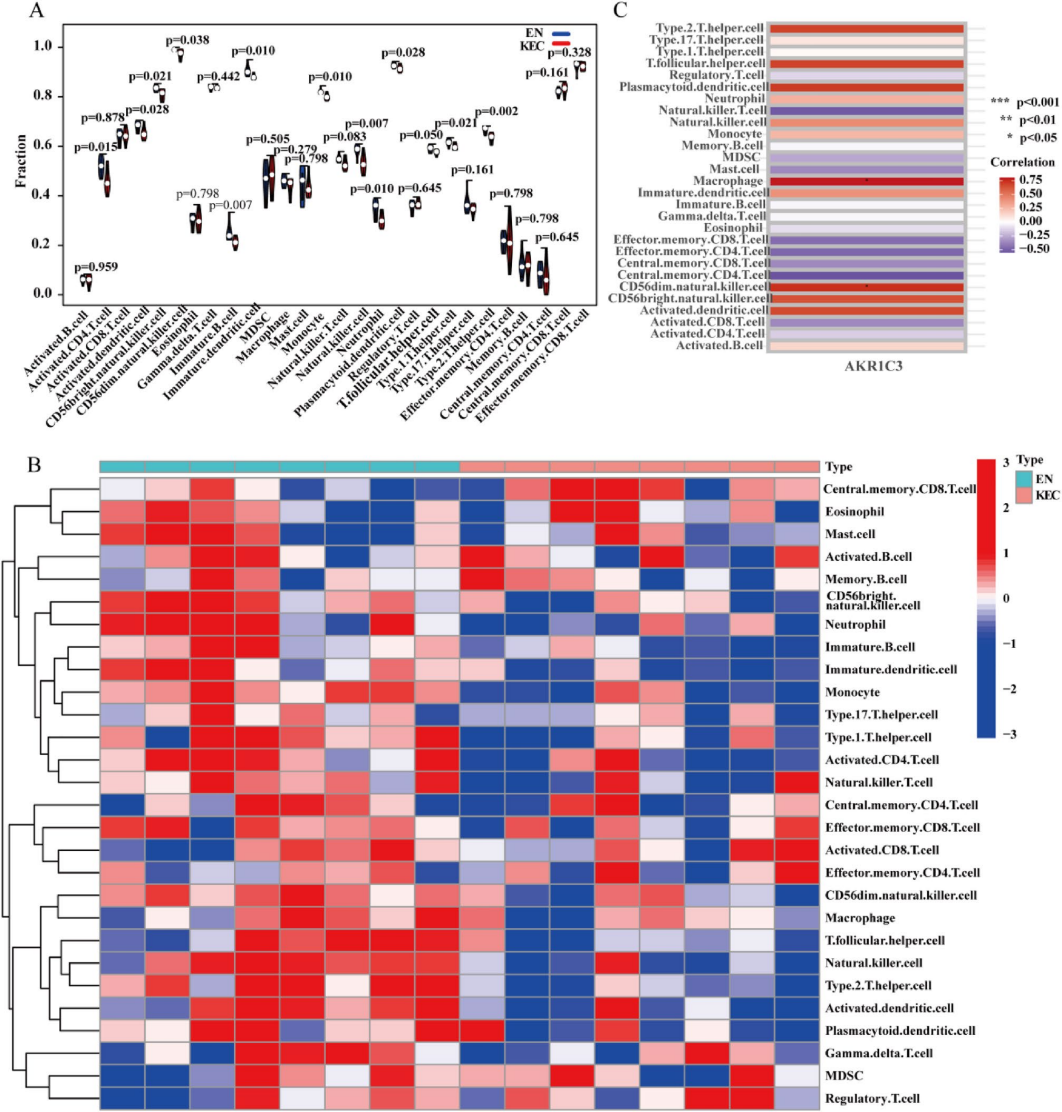
ssGSEA analysis. (**A**, **B**) Differential identification of immunophenotypic cells. (**C**) Immune subtype cells affected by AKR1C3. *p < 0.05.

In previous studies, from the perspective of data integrity analysis, KC was affected by inflammatory immune regulation, especially the Signaling by Interleukins. We performed Single Sample Gene Set Enrichment Analysis (ssGSEA) on the data by analyzing the association between samples and immune cells as a whole. Immune scoring of the samples was performed, and then visual analysis of the immune cell subsets of EKC and EN were performed to explore the different immune cell subsets in the samples. The results showed that keratoconus epithelial cells significantly affected immune subtype cells such as B cells, T cells, and natural killer cells (Fig. 7A,B). The immune cells regulated by AKR1C3 included CD56dim natural killer cells and macrophages (Fig. 7C). Similarly, immunophenotypic differences were observed between SN and SKC samples (Supplementary Fig. S7). These results suggested that KC may be affected by immune regulation.

### Gene-drug therapy and mRNA target prediction

**Figure 8:**
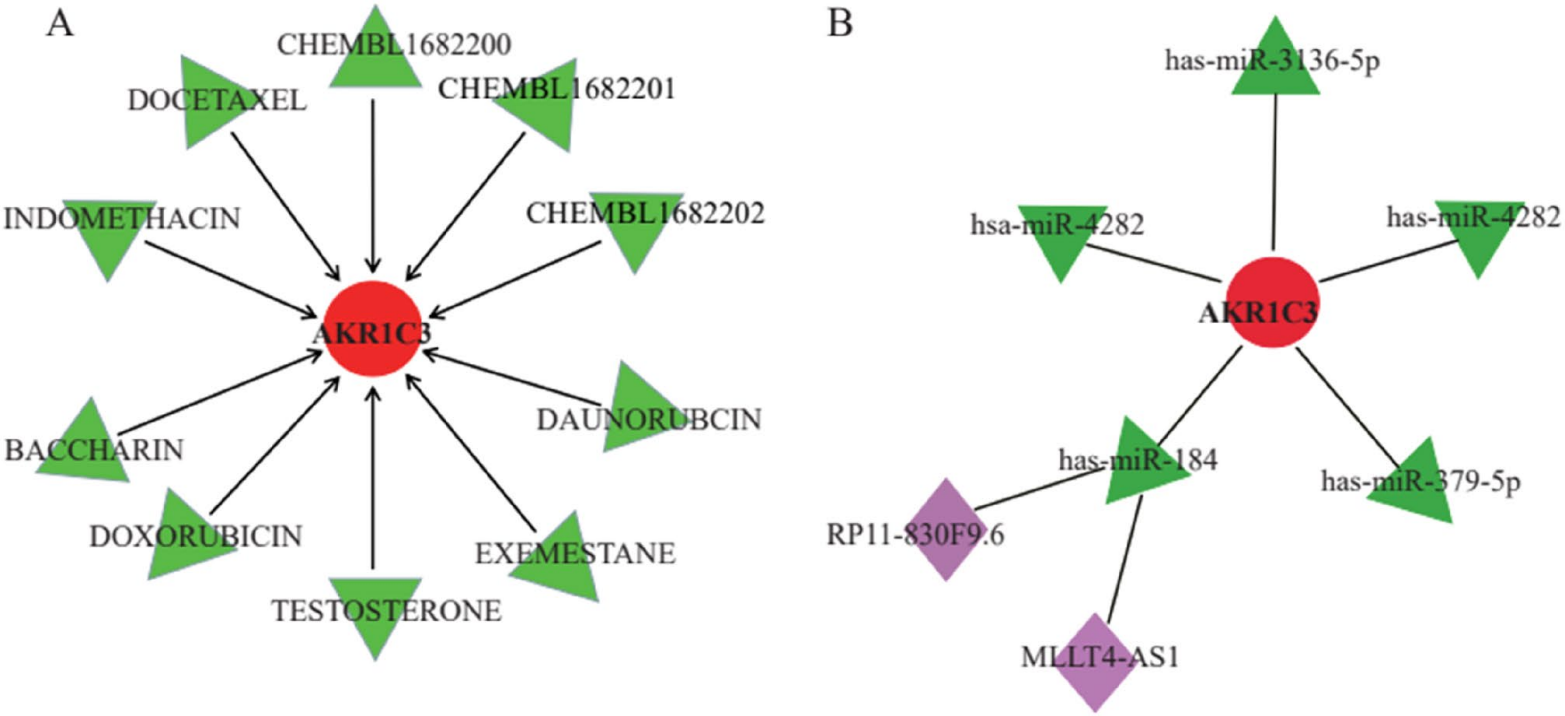
Gene-drug therapy and mRNA target prediction. (**A**) Prediction of gene-targeted drugs, using the website (https://dgidb.genome.wustl.edu/). (**B**) miRNA and IncRNA combined with characteristic genes.

The Drug and Gene Interaction website was used to predict drugs that interacted with AKR1C3 (https://dgidb.genome.wustl.edu/). The use of these drugs was based on previously reported studies that targeted AKR1C3 for gene therapy. After systematic screening, 10 drugs were identified (DAUNORUBICN, EXEMESTANE, TESTOSTERONE, DOXORUBICIN, BACCHARIN, INDOMETHACIN, DOCETAXEL, CHEMBL1682202, CHEMBL1682201, CHEMBL1682200). Among them, INDOMETHACIN, DAUNORUBICIN, DOXORUBICIN and DOCETAXEL exerted therapeutic effects by immunotherapeutically targeting AKR1C3 (Fig. 8A). Next, we predicted miRNA binding to AKR1C3 through miRNA databases (miRanda, miRDB, TargetScan), and the prediction results needed to satisfy the three databases at the same time. Five miRNAs satisfied the conditions (hsa-miR-3136-5p, hsa-miR-4282, hsa-miR-184, hsa-miR-3176, hsa-miR-379-5p) (Fig. 8B). We found that hsa-miR-184 was directly correlated with AKR1C3, miR-184 was the most abundant miRNA expressed in the cornea, which is related to the regulation of protein levels in the cornea. Next, we predicted the IncRNAs that bind to has-miR-184: RP11-830F9.6 and MLLT4-AS1. Therefore, we speculated that hsa-miR-184 may be an effective target for the treatment of KC and exert its effect through immunization.

## Discussion

KC is characterized by corneal thinning, central protrusion, and irregular astigmatism, resulting in serious compromise of visual acuity. The most widely accepted etiologies of keratoconus have been ascribed to genetic and environmental factors; it has been stated that KC is well known as an independent symptom that does not involve any systemic or ocular disease^11^, and that KC is a noninflammatory corneal disease^29^. Treatments of keratoconus have principally relied on corneal transplantation^30^ and corneal cross-linking^31^. However, a growing body of evidence suggests that the progression of KC involves the up-regulation of proinflammatory mediators and the involvement of Toll-like 2/4 receptors, which are influenced by the immune system^32,33^. The ocular surface of patients with KC has been clearly shown to demonstrate immune-inflammatory features^13^. Moreover, large amounts of superoxide, hydrogen peroxide and hydroxyl radicals have been reported on the ocular surface of keratoconus patients^34,35^. The oxidative stress response induced by environmental changes in KC is the direct cause of increased ROS levels and decreased antioxidant levels in keratoconus cells, which ultimately are responsible for extracellular matrix degradation and subsequent corneal stromal thinning^16,36^.

Ferroptosis is a unique, iron-dependent, non-apoptotic form of cell death. The accumulation of iron-dependent lipid peroxidation is mainly triggered by the Fenton reaction and lipoxygenases. Ferroptosis can affect the inflammatory response of cells through immunogenicity, and inhibition of ferroptosis can effectively suppress the inflammatory response; ferroptosis inhibitors have been shown to play important roles in several diseases through their anti-inflammatory effects^37^. Evidence of inflammation in ferroptosis has been reported, and the infiltration of neutrophils and the expression of pro-inflammatory cytokines were inhibited by Fer-1^38^. It is logical, therefore, that ferroptosis may play an important role in KC, but prior research supporting this concept has been lacking. In the present study, we elucidated the relationship between KC, ferroptosis and the immune microenvironment for the first time, which could reasonably provide new clues for the molecular mechanism of KC.

In this study, we used the data from GSE204791 to identify mRNAs associated with ferroptosis, extracted KEC and EN data for analysis, using the SKC and SN for study verification. Then we found that cell-receptor interaction, inflammation, and ferroptosis genes were significantly enriched when GSEA was used to analyze RNA-seq data. Notably, certain immune and inflammatory factors have previously been detected on the ocular surface of SK patients, as well as products of mtDNA damage in the KC cornea^39^. In our study, excessive ROS and RNS, disruption of antioxidant machinery, and mitochondrial dysfunction were found in KCS, suggesting that increased oxidative stress played an important role in the pathogenesis of KCS^40^. Therefore, we postulated that keratoconus and ferroptosis were significantly associated. With further pursuit using WGCNA analysis, genes were classified, and 8 genes related to ferroptosis were screened, and GO analysis demonstrated that most of them (NOS2, AKR1C3, CAV1, SNCA, SLC7A11) were closely related to oxidative stress and iron ion transport. Then, hub genes were screened by SVM-RFE, and 9 genes (AQP5, ATF2, ARNTL, AKR1C3, FXN, MGST1, ARNT, LCN2, SLC11A2, SMPD1) related to oxidative stress and metal ion transport were analyzed by GO. Finally, the two gene sets were intersected and AKR1C3 came to the fore.

AKR1C3, a member of the aldosterone-reductase (AKR) family, is a hormone activity regulator with various cellular functions, including the regulation of prostaglandin, steroid hormone, and retinoid metabolism^41^. AKR1C3 has been reported to have oxidoreductase activity and AKR (NADPH) activity^42^, and is involved in the regulation of cellular redox homeostasis and immunity of tumor cells^43^. As an inhibitor of ferroptosis, the reduction of AKR1C3 protein expression can stimulate the occurrence of ferroptosis^44^. The cystine/glutamate antiporter SLC7A11 (commonly known as xCT) can import cystine for glutathione biosynthesis and antioxidant defense, and high expression of SLC7A11 can effectively suppress ferroptosis, which has been clearly verified in many cancers^45,46^. However, studies have shown that overexpression of AKR1C3 can promote the increase of SLC7A11 in HCC cells^44^. In our analysis, SLC7A11 expression was significantly reduced, which may have been caused by the reduction of AKR1C3, which in turn inhibited the xCT system and promoted ferroptosis. Genetics has demonstrated that maintaining GSH synthesis or promoting the activity of the system xCT protects cells from death triggered by oxidative stress conditions^47,48^. Similarly, the accumulation of reactive oxygen species (ROS) is an important mechanism that causes oxidative stress death of endothelial cells, and ROS can promote cell death through ferroptosis^49^; this seems to provide strong support for ROS accumulation in keratoconus. Similarly, evidence suggests that ferroptosis plays an important role in immune cell-induced cell death^50^. For example, NK cells have reportedly been involved in ferroptosis, lipid peroxidation and increased expression of proteins related to oxidative damage in tumor cells^51^, and macrophages showed resistance to ferroptosis^52^.

Prior reports have indicated that miR-184 is the most abundant miRNA that is expressed in corneal and lens epithelial cells, which is associated with the horizontal adjustment of proteins in the cornea^53,54^. In the present study, AKR1C3 was found to be responsible for the synthesis of miR-184, and the expression of AKR1C3 was decreased in keratoconus. Therefore, the synthesis of miR-184, which is responsible for the regulation of protein level, was also impaired, likely an important reason affecting KC. We also predicted IncRNA (MLLT4-AS1 and RP11-830F9.6), associated with miR-184 synthesis, which is essential for the molecular function of miR-184. The ultimate aim was to predict the corresponding immune drugs, which could serve in interfering with keratoconus at the genetic level. Of the 10 drugs predicted to target AKR1C3, 4 (INDOMETHACIN, DAUNORUBICIN, DOXORUBICIN, DOCETAXEL) were selected, principally because they target AKR1C3. Unfortunately, all except Indomethacin are confined to the treatment of tumors. Nevertheless, drug development aimed at the treatment of genes is still in its relative infancy.

In conclusion, our study revealed that the pathogenesis of keratoconus may be due at least in part to ferroptosis on the ocular surface, and the core gene AKR1C3 was identified by bioinformatics analysis, showed a connection to the expression of miR-184 and other miRNAs, as well as the over expression of inflammatory factors and immune subtype cells.

### Supplementary Information

Supplementary Information.

## Data Availability

The datasets generated or analyzed during the current study are freely available for the research community to access from the GEO (GSE204791) repository (https://www.ncbi.nlm.nih.gov/geo/query/acc.cgi?acc=GSE204791).

## References

[1] Lasagni Vitar RM, Bonelli F, Rama P, Ferrari G (2022). Nutritional and metabolic imbalance in keratoconus. Nutrients.

[2] Gordon-Shaag A, Millodot M, Shneor E, Liu Y (2015). The genetic and environmental factors for keratoconus. Biomed. Res. Int..

[3] Anitha V, Vanathi M, Raghavan A, Rajaraman R, Ravindran M, Tandon R (2021). Pediatric keratoconus—current perspectives and clinical challenges. Indian J. Ophthalmol..

[4] Galvis V, Sherwin T, Tello A, Merayo J, Barrera R, Acera A (2015). Keratoconus: An inflammatory disorder?. Eye (Lond.).

[5] Rabinowitz YS, Galvis V, Tello A, Rueda D, Garcia JD (2021). Genetics vs chronic corneal mechanical trauma in the etiology of keratoconus. Exp. Eye Res..

[6] Lucas SEM, Burdon KP (2020). Genetic and environmental risk factors for keratoconus. Annu. Rev. Vis. Sci..

[7] Dapena I, Parker JS, Melles GRJ (2020). Potential benefits of modified corneal tissue grafts for keratoconus: Bowman layer 'inlay' and 'onlay' transplantation, and allogenic tissue ring segments. Curr. Opin. Ophthalmol..

[8] Dragnea DC, Birbal RS, Ham L, Dapena I, Oellerich S, van Dijk K, Melles GRJ (2018). Bowman layer transplantation in the treatment of keratoconus. Eye Vis. (Lond.).

[9] Yang Q, Wang S, He Y, Zhang Y (2023). The research progress on the molecular mechanism of corneal cross-linking in keratoconus treatment. Contact Lens Anterior Eye.

[10] Bozkurt E, Ucak T (2021). Serum inflammation biomarkers in patients with keratoconus. Ocul. Immunol. Inflamm..

[11] Claessens JLJ, Godefrooij DA, Vink G, Frank LE, Wisse RPL (2022). Nationwide epidemiological approach to identify associations between keratoconus and immune-mediated diseases. Br. J. Ophthalmol..

[12] Navel V, Malecaze J, Pereira B, Baker JS, Malecaze F, Sapin V, Chiambaretta F, Dutheil F (2021). Oxidative and antioxidative stress markers in keratoconus: A systematic review and meta-analysis. Acta Ophthalmol..

[13] D'Souza S, Nair AP, Sahu GR, Vaidya T, Shetty R, Khamar P, Mullick R, Gupta S, Dickman MM, Nuijts R, Mohan RR, Ghosh A, Sethu S (2021). Keratoconus patients exhibit a distinct ocular surface immune cell and inflammatory profile. Sci. Rep..

[14] Yildiz E, Aydemir D, Zibandeh N, Kusan E, Gumus K, Ilhan Sarac O, Karslioglu MZ, Cagil N, Sahin A (2022). Investigation of mitophagy biomarkers in corneal epithelium of keratoconus patients. Curr. Eye Res..

[15] Li J, Cao F, Yin HL, Huang ZJ, Lin ZT, Mao N, Sun B, Wang G (2020). Ferroptosis: Past, present and future. Cell Death Dis..

[16] Park E, Chung SW (2019). ROS-mediated autophagy increases intracellular iron levels and ferroptosis by ferritin and transferrin receptor regulation. Cell Death Dis..

[17] Tang D, Chen X, Kang R, Kroemer G (2021). Ferroptosis: Molecular mechanisms and health implications. Cell Res..

[18] Chen X, Kang R, Kroemer G, Tang D (2021). Ferroptosis in infection, inflammation, and immunity. J. Exp. Med..

[19] Battaglia AM, Chirillo R, Aversa I, Sacco A, Costanzo F, Biamonte F (2020). Ferroptosis and cancer: Mitochondria meet the "iron maiden" cell death. Cells.

[20] Antoszczak M, Muller S, Caneque T, Colombeau L, Dusetti N, Santofimia-Castano P, Gaillet C, Puisieux A, Iovanna JL, Rodriguez R (2022). Iron-sensitive prodrugs that trigger active ferroptosis in drug-tolerant pancreatic cancer cells. J. Am. Chem. Soc..

[21] Gu R, Xia Y, Li P, Zou D, Lu K, Ren L, Zhang H, Sun Z (2022). Ferroptosis and its role in gastric cancer. Front. Cell. Dev. Biol..

[22] Changshun, Y. *et al.* Suppressing the KIF20A/NUAK1/Nrf2/GPX4 signaling path reduces ferroptosis and enhances the sensitivity of colored cancer to oxaliplatin. *Aging* **13**(10), 13515–13534 (2021). 10.18632/aging.202774 PMC8202845 33819186

[23] Li S, Zheng L, Zhang J, Liu X, Wu Z (2021). Inhibition of ferroptosis by up-regulating Nrf2 delayed the progression of diabetic nephropathy. Free Radic. Biol. Med..

[24] Mu L, Wang D, Dong Z, Wu J, Wu X, Su J, Zhang Y (2022). Abnormal levels of serum ferroptosis-related biomarkers in diabetic retinopathy. J. Ophthalmol..

[25] Guo Y, Zhang W, Zhou X, Zhao S, Wang J, Guo Y, Liao Y, Lu H, Liu J, Cai Y, Wu J, Shen M (2022). Roles of ferroptosis in cardiovascular diseases. Front. Cardiovasc. Med..

[26] Buddi R, Lin B, Atilano SR, Zorapapel NC, Kenney MC, Brown DJ (2002). Evidence of oxidative stress in human corneal diseases. J. Histochem. Cytochem..

[27] Hao XD, Chen ZL, Qu ML, Zhao XW, Li SX, Chen P (2016). Decreased integrity, content, and increased transcript level of mitochondrial DNA are associated with keratoconus. PLoS ONE.

[28] Cai J, Estes A, Liu Y (2020). Omics analyses in keratoconus: From transcriptomics to proteomics. Curr. Ophthalmol. Rep..

[29] Krachmer JH, Feder RS, Belin MW (1984). Keratoconus and related noninflammatory corneal thinning disorders. Surv. Ophthalmol..

[30] Hwang S, Chung TY, Han J, Kim K, Lim DH (2021). Corneal transplantation for keratoconus in South Korea. Sci. Rep..

[31] Bikbova G, Kazakbaeva G, Bikbov M, Usubov E (2018). Complete corneal ring (MyoRing) implantation versus MyoRing implantation combined with corneal collagen crosslinking for keratoconus: 3-year follow-up. Int. Ophthalmol..

[32] Sun X, Zhang H, Shan M, Dong Y, Zhang L, Chen L, Wang Y (2022). Comprehensive transcriptome analysis of patients with keratoconus highlights the regulation of immune responses and inflammatory processes. Front. Genet..

[33] McKay TB, Serjersen H, Hjortdal J, Zieske JD, Karamichos D (2020). Characterization of tear immunoglobulins in a small-cohort of keratoconus patients. Sci. Rep..

[34] Shoham A, Hadziahmetovic M, Dunaief JL, Mydlarski MB, Schipper HM (2008). Oxidative stress in diseases of the human cornea. Free Radic. Biol. Med..

[35] Behndig A, Karlsson K, Johansson BO, Brannstrom T, Marklund SL (2001). Superoxide dismutase isoenzymes in the normal and diseased human cornea. Invest. Ophthalmol. Vis. Sci..

[36] Arnal E, Peris-Martinez C, Menezo JL, Johnsen-Soriano S, Romero FJ (2011). Oxidative stress in keratoconus?. Invest. Ophthalmol. Vis. Sci..

[37] Sun Y, Chen P, Zhai B, Zhang M, Xiang Y, Fang J, Xu S, Gao Y, Chen X, Sui X, Li G (2020). The emerging role of ferroptosis in inflammation. Biomed. Pharmacother..

[38] Linkermann A, Skouta R, Himmerkus N, Mulay SR, Dewitz C, De Zen F, Prokai A, Zuchtriegel G, Krombach F, Welz PS, Weinlich R, Vanden Berghe T, Vandenabeele P, Pasparakis M, Bleich M, Weinberg JM, Reichel CA, Brasen JH, Kunzendorf U, Anders HJ, Stockwell BR, Green DR, Krautwald S (2014). Synchronized renal tubular cell death involves ferroptosis. Proc. Natl. Acad. Sci. U. S. A..

[39] Atilano SR, Coskun P, Chwa M, Jordan N, Reddy V, Le K, Wallace DC, Kenney MC (2005). Accumulation of mitochondrial DNA damage in keratoconus corneas. Invest. Ophthalmol. Vis. Sci..

[40] Wisse RPL, Kuiper JJW, Radstake TRD, Broen JCA (2019). Quantification of double stranded DNA breaks and telomere length as proxies for corneal damage and replicative stress in human keratoconus corneas. Transl. Vis. Sci. Technol..

[41] Liu Y, He S, Chen Y, Liu Y, Feng F, Liu W, Guo Q, Zhao L, Sun H (2020). Overview of AKR1C3: Inhibitor achievements and disease insights. J. Med. Chem..

[42] Zhou C, Wang Z, Li J, Wu X, Fan N, Li D, Liu F, Plum PS, Hoppe S, Hillmer AM, Quaas A, Gebauer F, Chon SH, Bruns CJ, Zhao Y (2021). Aldo–keto reductase 1C3 mediates chemotherapy resistance in esophageal adenocarcinoma via ROS detoxification. Cancers (Basel).

[43] Cui X, Li C, Ding J, Yao Z, Zhao T, Guo J, Wang Y, Li J (2023). Establishing a proteomics-based signature of AKR1C3-related genes for predicting the prognosis of prostate cancer. Int. J. Mol. Sci..

[44] Chen J, Zhang J, Tian W, Ge C, Su Y, Li J, Tian H (2023). AKR1C3 suppresses ferroptosis in hepatocellular carcinoma through regulation of YAP/SLC7A11 signaling pathway. Mol. Carcinog..

[45] Koppula P, Zhuang L, Gan B (2021). Cystine transporter SLC7A11/xCT in cancer: Ferroptosis, nutrient dependency, and cancer therapy. Protein Cell.

[46] Yang J, Zhou Y, Xie S, Wang J, Li Z, Chen L, Mao M, Chen C, Huang A, Chen Y, Zhang X, Khan NUH, Wang L, Zhou J (2021). Metformin induces ferroptosis by inhibiting UFMylation of SLC7A11 in breast cancer. J. Exp. Clin. Cancer Res..

[47] Fang X, Cai Z, Wang H, Han D, Cheng Q, Zhang P, Gao F, Yu Y, Song Z, Wu Q, An P, Huang S, Pan J, Chen HZ, Chen J, Linkermann A, Min J, Wang F (2020). Loss of cardiac ferritin H facilitates cardiomyopathy via Slc7a11-mediated ferroptosis. Circ. Res..

[48] Mandal PK, Seiler A, Perisic T, Kolle P, Banjac Canak A, Forster H, Weiss N, Kremmer E, Lieberman MW, Bannai S, Kuhlencordt P, Sato H, Bornkamm GW, Conrad M (2010). System x(c)- and thioredoxin reductase 1 cooperatively rescue glutathione deficiency. J. Biol. Chem..

[49] Zheng D, Liu J, Piao H, Zhu Z, Wei R, Liu K (2022). ROS-triggered endothelial cell death mechanisms: Focus on pyroptosis, parthanatos, and ferroptosis. Front. Immunol..

[50] Jiang X, Stockwell BR, Conrad M (2021). Ferroptosis: Mechanisms, biology and role in disease. Nat. Rev. Mol. Cell Biol..

[51] Poznanski SM, Singh K, Ritchie TM, Aguiar JA, Fan IY, Portillo AL, Rojas EA, Vahedi F, El-Sayes A, Xing S, Butcher M, Lu Y, Doxey AC, Schertzer JD, Hirte HW, Ashkar AA (2021). Metabolic flexibility determines human NK cell functional fate in the tumor microenvironment. Cell Metab..

[52] Xia Y, Rao L, Yao H, Wang Z, Ning P, Chen X (2020). Engineering macrophages for cancer immunotherapy and drug delivery. Adv. Mater..

[53] Abu-Amero KK, Helwa I, Al-Muammar A, Strickland S, Hauser MA, Allingham RR, Liu Y (2015). Screening of the seed region of MIR184 in keratoconus patients from Saudi Arabia. Biomed. Res. Int..

[54] Bykhovskaya Y, Caiado Canedo AL, Wright KW, Rabinowitz YS (2015). C.57 C > T mutation in MIR 184 is responsible for congenital cataracts and corneal abnormalities in a five-generation family from Galicia, Spain. Ophthalmic Genet..

