# Supplementary material for: Screening and identification of genes related to ferroptosis in keratoconus
Source: Sci Rep. 2023 Aug 25;13:13956. doi: 10.1038/s41598-023-41194-2 (PMC10457308; doi:10.1038/s41598-023-41194-2)

**Screening and identification of genes related to ferroptosis in keratoconus**

Xiaojun Wu^1,2#^, Qing Deng^1,2#^, Zhe Han^1,2^, Feixue Ni^1,2^, Daxi Sun^1,2^, Yuxue Xu^1,2^

・Authors’ affiliations:

^1^School of Pharmacology, Binzhou Medical University, Yantai 264003, China

^2^Shandong Technology Innovation Center of Molecular Targeting and Intelligent Diagnosis and Treatment, Yantai 264003, China

・Corresponding author: Yuxue Xu, xuyuxue@bzmc.edu.cn. Binzhou Medical University, Guanhai Rd 346, Yantai, 264003, China.

・# Co-first authors: Xiaojun Wu and Qing Deng contributed equally to this work.

**Supplemental Figures**

**Supplemental table 1.** Results of partial GSEA enrichment analysis

| **GSEA description** | **enrichmentScore** | **NES** | **pvalue** | **p.adjust** |
| --- | --- | --- | --- | --- |
| NABA - Secreted - Factors | -0.49 | -1.82 | 3.3E-09 | 3.3E-09 |
| Cytokine - Cytokine Recepor Interaction | -0.51 | -1.84 | 5.5E-08 | 5.5E-08 |
| Chemokine Signaling Pathway | -0.53 | -1.88 | 6.2E-07 | 6.2E-07 |
| Overview of Proinflammatory and Profibrotic Mediators | -0.57 | -1.898 | 1.8E-06 | 1.8E-06 |
| Network Map of Sarscov2 Signaling Pathway | -0.50 | -1.78 | 1.9E-06 | 1.9E-06 |
| Reactome Chemokine Receptors Bind Chemokines | -0.70 | -2.02 | 2.3E-06 | 2.3E-06 |
| Reactome Signaling By Interleukins | -0.42 | -1.57 | 5.5E-06 | 5.5E-06 |
| Reactome Interleukin 10 Signaling | -0.70 | -1.97 | 1.5E-05 | 1.5E-05 |
| Chemokine Signaling Pathway | -0.51 | -1.76 | 1.8E-05 | 1.8E-05 |
| Sarscov2 Innate Evasion and Cellspecific Immunity Response | -0.62 | -1.90 | 4.1E-05 | 4.1E-05 |
| Ferroptosis | -0.54 | -1.63 | 0.0040 | 0.0040 |
| Reactome Extracellular Matrix Organization | -0.43 | -1.59 | 4.1E-05 | 4.1E-05 |
| TGF B Receptor Signaling In Skeletal Dysplasias | -0.64 | -1.90 | 4.2E-05 | 4.2E-05 |
| Vitamin D Receptor Pathway | -0.48 | -1.69 | 5.4E-05 | 5.4E-05 |
| TGF B Receptor Signaling | -0.64 | -1.86 | 7.3E-05 | 7.3E-05 |
| Type Ⅱ Interferon Signaling | -0.71 | -1.91 | 7.6E-05 | 7.6E-05 |
| TYROBP Causal Network In Microglia | -0.63 | -1.87 | 7.7E-05 | 7.7E-05 |
| PID IL23 Pathway | -0.68 | -1.86 | 1.2E-04 | 1.2E-04 |
| Reactome Rho GTPase Cycle | -0.39 | -1.47 | 1.8E-04 | 1.8E-04 |
| Fibrin Complement Receptor 3 Signaling Pathway | -0.66 | -1.85 | 1.8E-04 | 1.8E-04 |

**Supplementary Figure S1****. Validation of EN and EKC samples from GSE204791.** (A) The blue boxes of the box-plot represents EN samples, and the red boxes represents KEC samples. The black lines represent the sample median, and its distribution represents the degree of sample standardization. (B) Visualization of principal component analysis of EN and EKC. (C) Cluster dendrogram. The result represents the correlation between the samples. (D) Volcano plot of differentially expressed genes. (| Log_2_FC (Fold change) | ≥ 0.58) represents a 0.5 times difference between the experimental group and the control group.


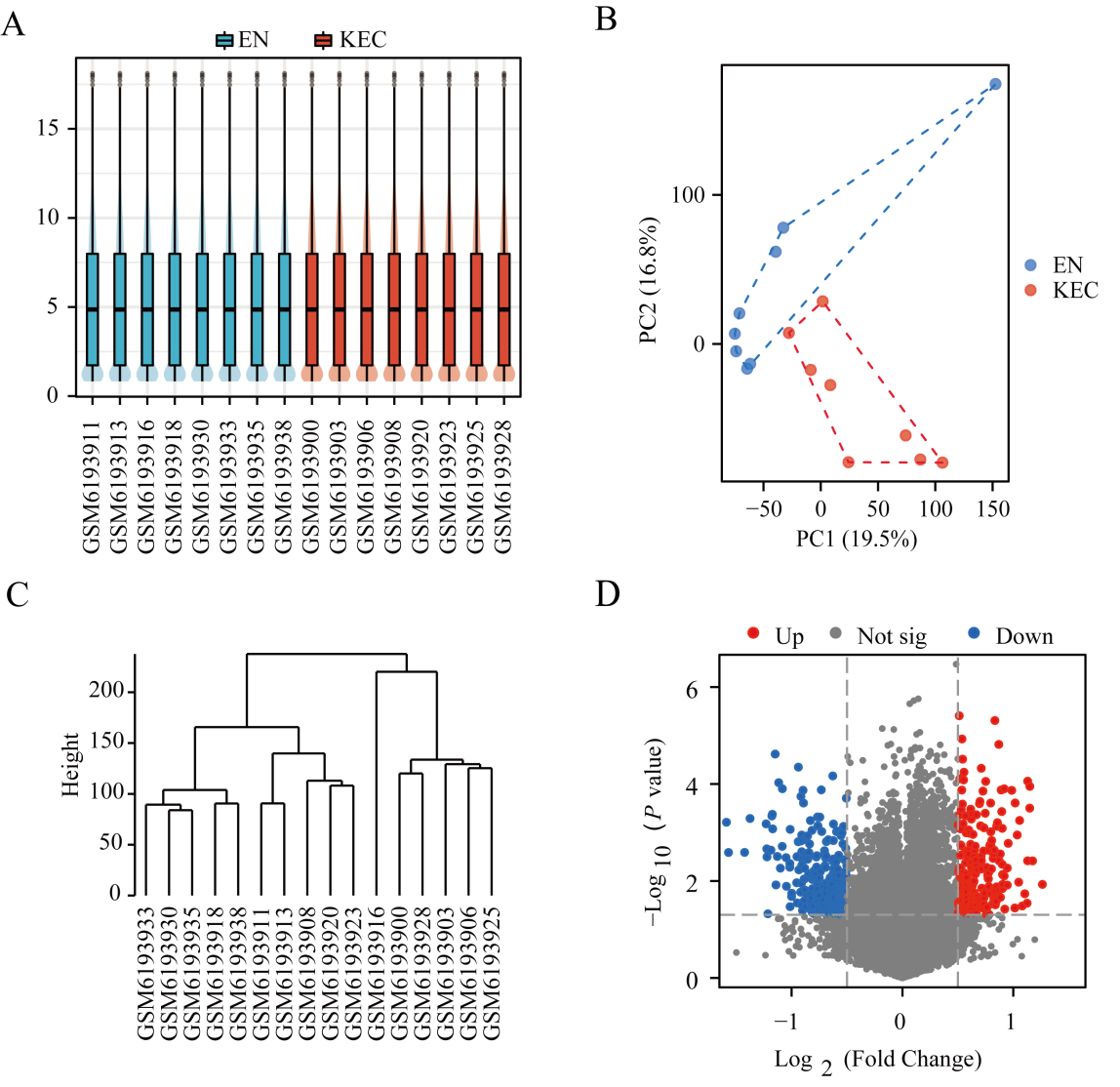


**Supplementary Figure S2. Validation of SN and SKC samples from GEO204791.** (A) The blue boxes of the box-plot represents SN samples, and the red boxes represents SKC samples. The black lines represent the sample median, and its distribution represents the degree of sample standardization. (B) Visualization of principal component analysis of SN and SKC.


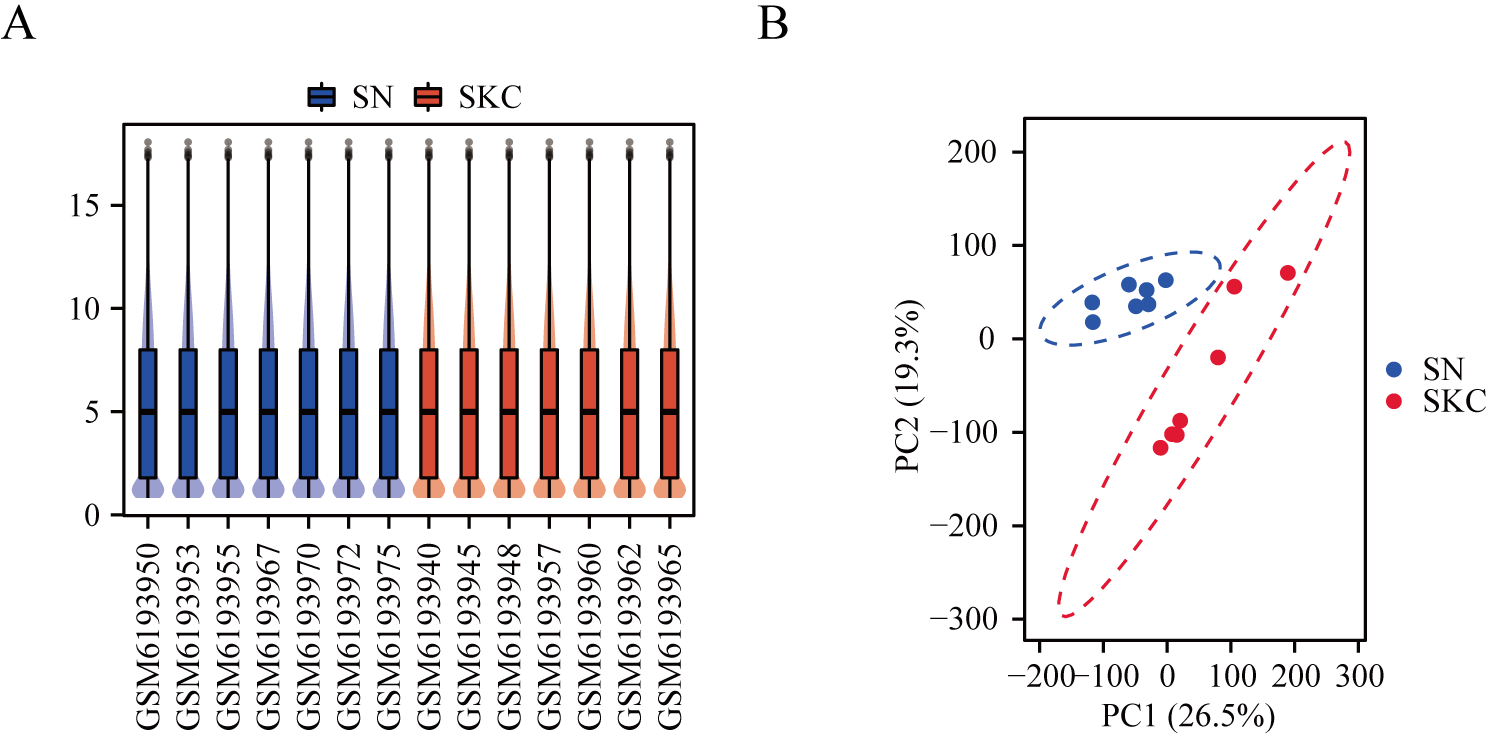


**Supplementary Figure S3. Representative results of GSEA analysis in the expression data of SN and SKC.** (A) A significant gene set negatively correlated with SKC group was Cytokine Cytokine Receptor Interaction(NES=-2.240, P.adj＜0.001). (B) Signaling by Interleukins was negatively correlated with EKC(NES=-2.209, P.adj＜0.001). (C) Ferroptosis-related genes were negatively correlated with keratoconus (NES=-1.757, P.adj=0.022). NES, normalized enrichment score.


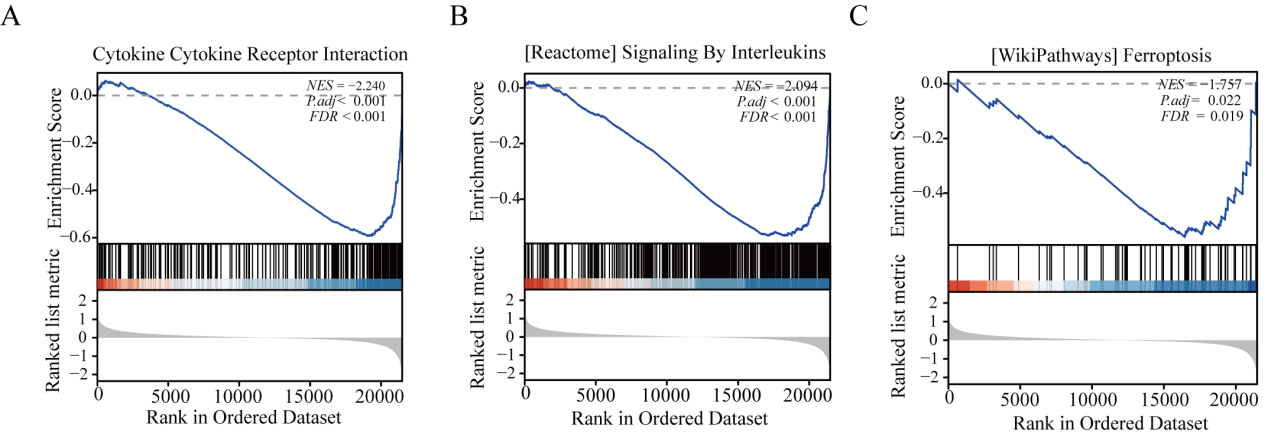


**Supplementary Figure S4. A co-expression network was established using WGCNA.** (A) module gene hierarchical clustering. Represent the Pearson correlation coefficient clustering between the samples, showing the relationship between the samples. (B and C) the topological relationship of the scale-free network of sample soft thresholds and the average connectivity of the power of various soft thresholds. (D) The cluster dendrogram of genes. (E) Module diagram of genes represented by different colors. (F) Heat map of adjacency relationships in the hub gene network.


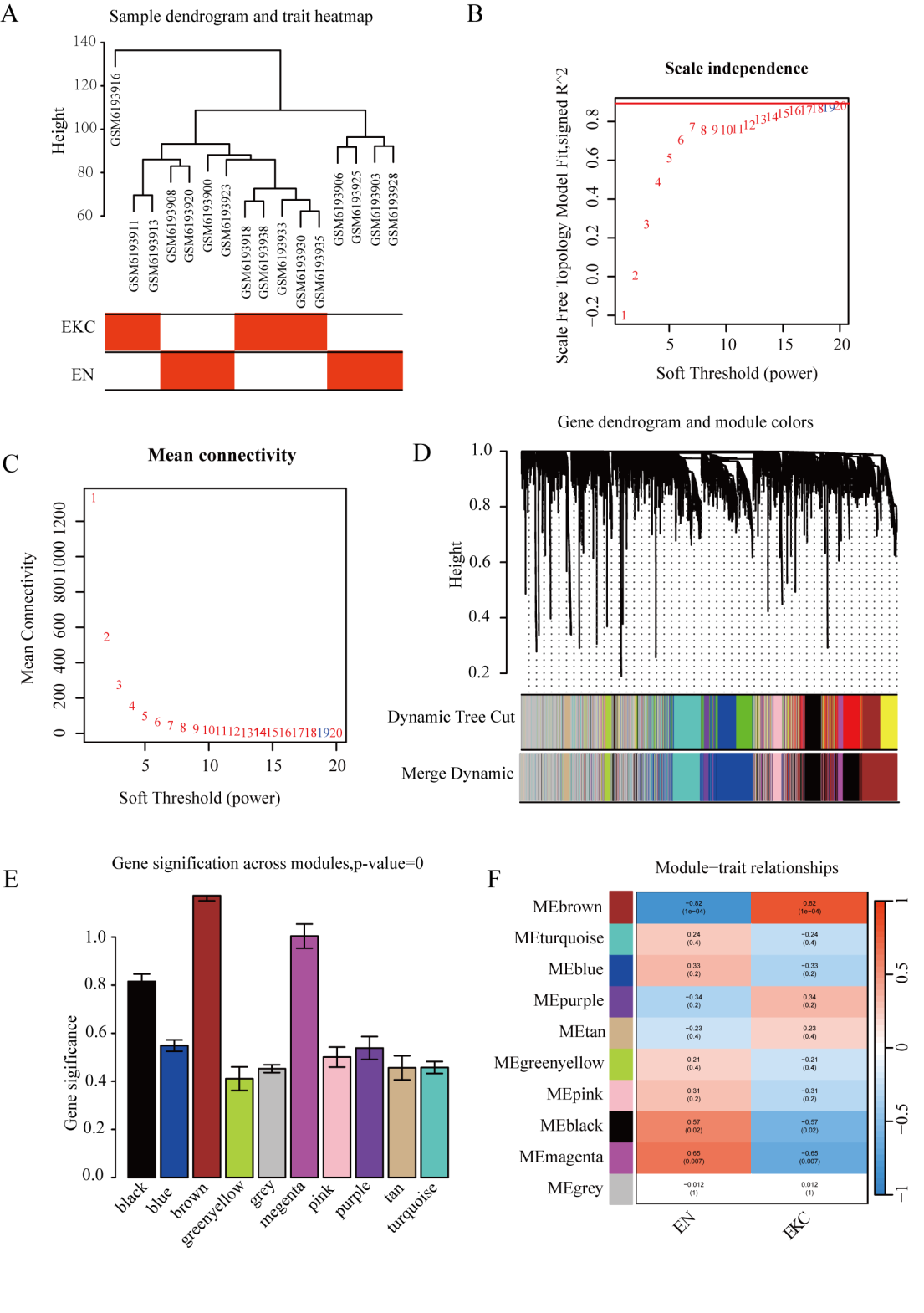


**Supplementary Figure S5. The heat map showed a total of 89 differential genes associated with ferroptosis**

**
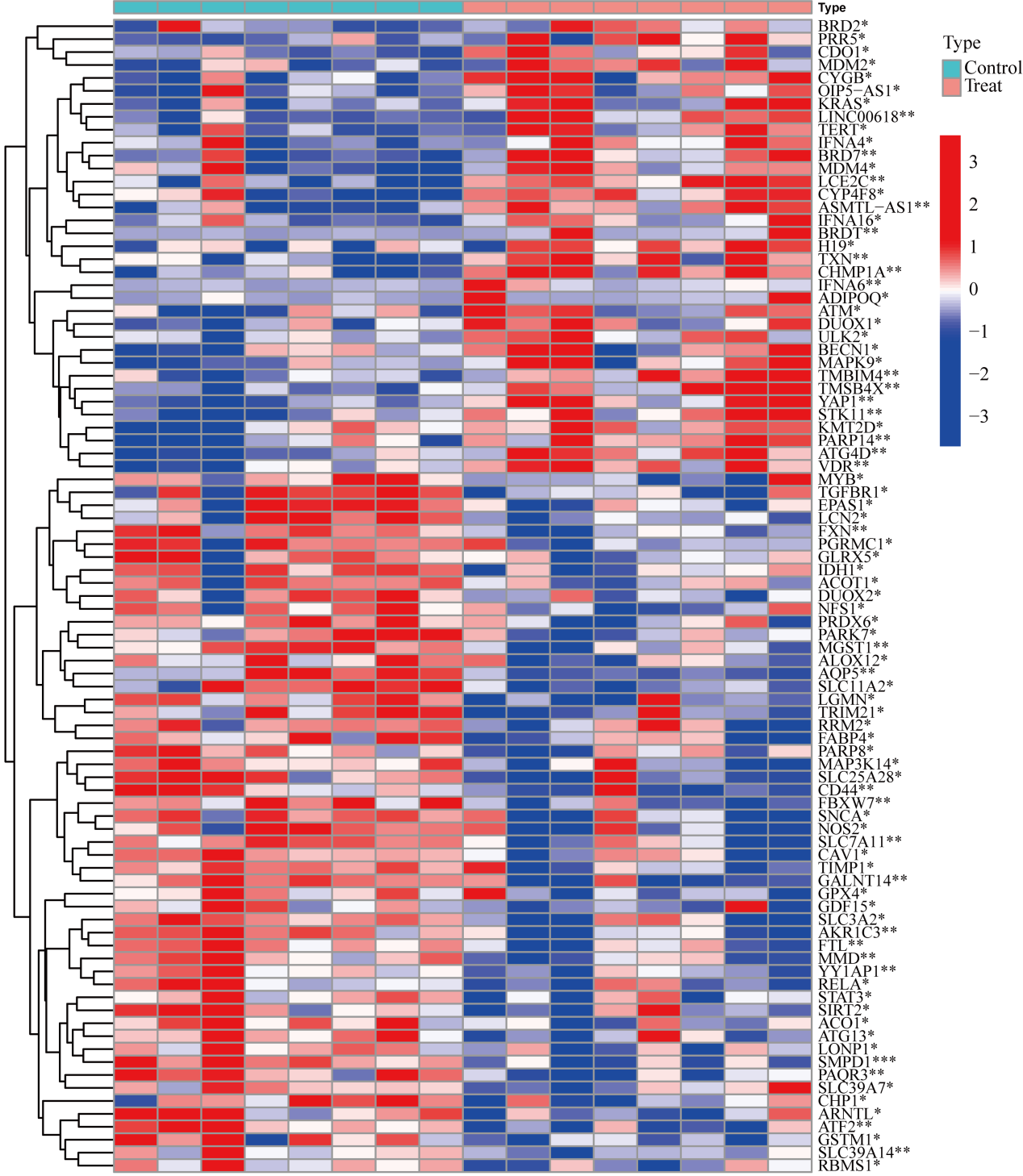
**

**Supplementary Figure S6. The differential gene expression and receiver operating characteristic analysis (ROC).** (A) The mRNA expression of AKR1C3. (B) The mRNA expression of SLC7A11. (C) The AUC analysis of AKR1C3. Receiver operating characteristic, ROC; AUC, area under the ROC curve.


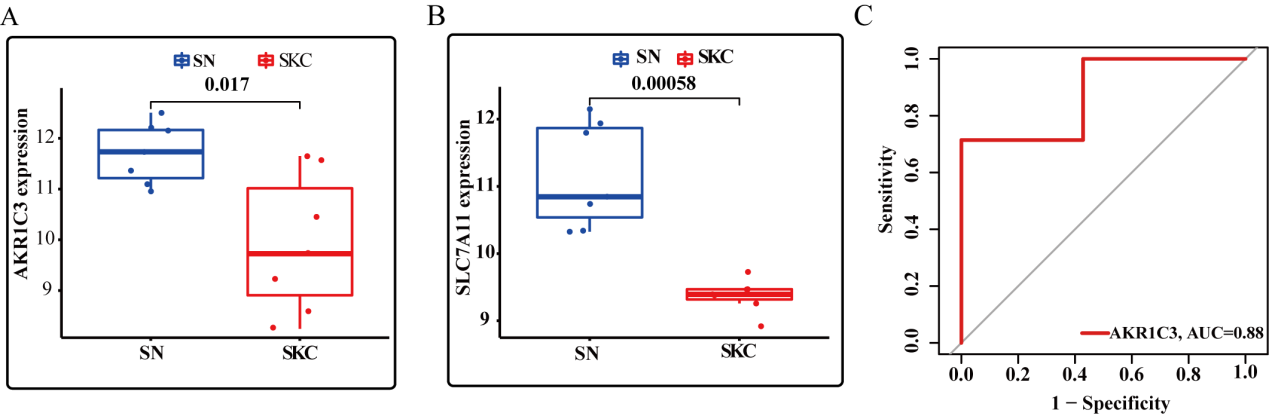


**Supplementary Figure S7. ssGSEA analysis.** (A and B) Differential identification of immunophenotypic cells. (C) Immune subtype cells affected by AKR1C3. *p＜0.05.


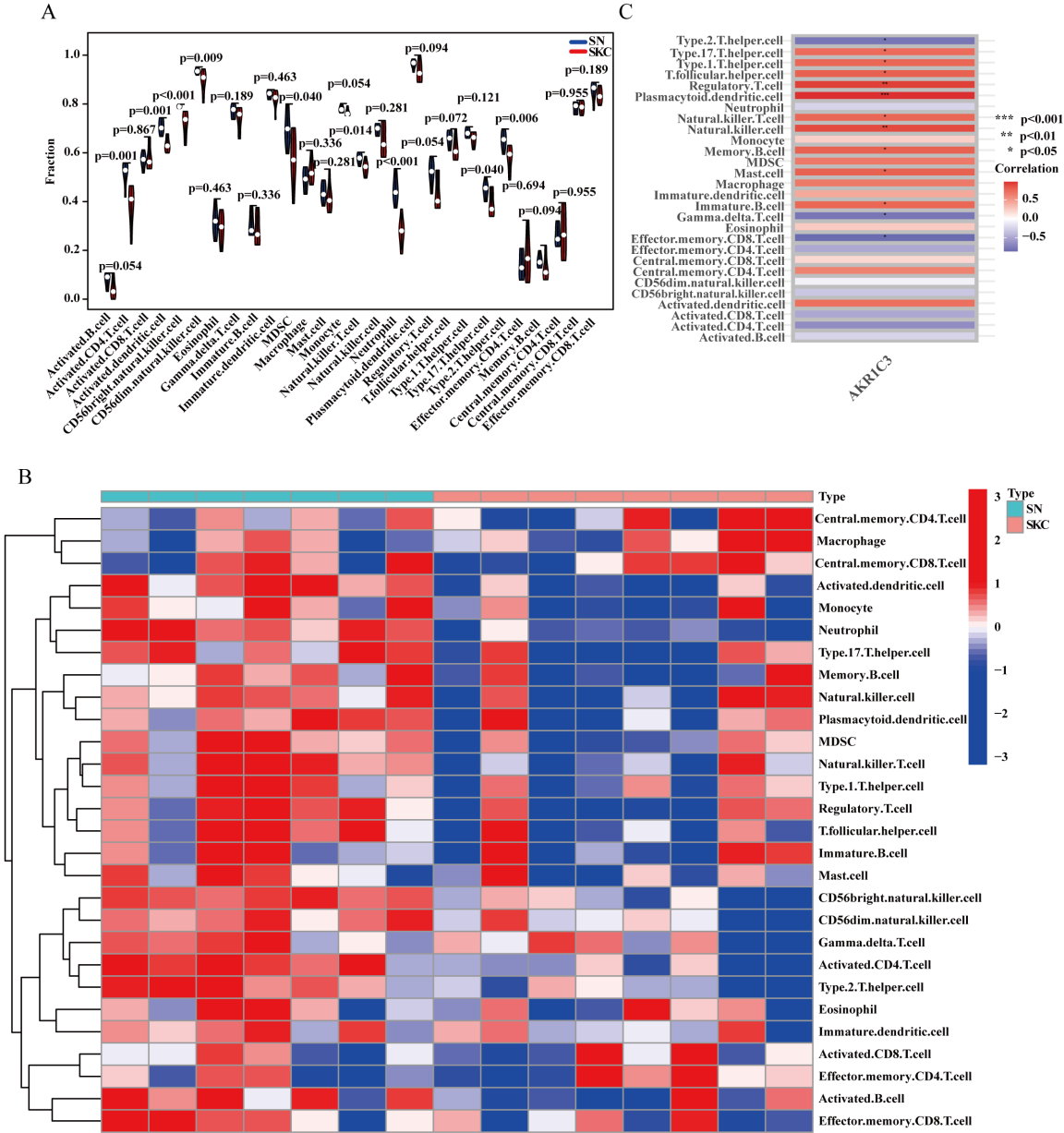

Supplement: Supplementary file 1 — Supplementary Information. [file 41598_2023_41194_MOESM1_ESM.docx]
